# Correlation of metallothionein expression with apoptosis in nasopharyngeal carcinoma

**DOI:** 10.1054/bjoc.1999.1063

**Published:** 2000-02-21

**Authors:** A Jayasurya, B H Bay, W M Yap, N G Tan

**Affiliations:** Department of Anatomy, Faculty of Medicine, National University of Singapore, Kent Ridge, S119260, Singapore; Department of Pathology, Tan Tock Seng Hospital, Jalan Tan Tock Seng, S308433, Singapore; Department of Otolaryngology, Singapore General Hospital, Outram Road, S169608, Singapore

**Keywords:** nasopharyngeal cancer, metallothionein immunohistochemistry, nuclear localization, apoptosis, ultrastructure

## Abstract

The expression of metallothionein (MT), an intracellular ubiquitous low molecular weight protein thiol with antioxidant properties, was studied in nasopharyngeal cancer (NPC) and correlated with the apoptotic index. Immunohistochemical staining of randomly selected, formalin-fixed and paraffin-embedded normal and malignant nasopharyngeal tissues were analysed for the expression of MT using the commercially available E9 antibody directed against MT I and MT II isoforms. The corresponding apoptosis labelling indices were evaluated by the TUNEL method. Localization of MT at the ultrastructural level was studied by immunogold labelling. All the tumour sections (17 specimens) showed MT-immunopositivity. A direct correlation between the percentage of MT-positive cells and the staining intensity was noted (*P*< 0.001; Pearson's *r* = 0.95). There was absence of cytoplasmic staining and only nuclear staining (with localization in the nucleoplasm) was demonstrated in the tumour cells. In normal epithelium of the nasopharynx, the basal layer was stained. An inverse relationship was observed between the level of MT expression and the apoptotic index in the NPC tissues (*P* = 0.0059; Pearson's *r* = –0.6380). The results suggest that overexpression of MT in NPC may protect the tumour cells from entering into the apoptotic process and thereby contribute to tumour expansion. Preferential localization of MT in the nuclei of NPC cells may possibly enhance radioresistance since radiotherapy is known to eradicate tumour cells by free radical-induced apoptosis. © 2000 Cancer Research Campaign


*British Journal of Cancer* (2000) **82**(6), 1198–1203
© 2000 Cancer Research Campaign
Article no. bjoc.1999.1063, available online at http://www.idealibrary.com on IDEAL®

### Correlation of metallothionein expression with apoptosis in nasopharyngeal carcinoma

**A Jayasurya[1], BH Bay[1], WM Yap[2] and NG Tan[3]**

[1]Department of Anatomy, Faculty of Medicine, National University of Singapore, Kent Ridge, S119260, Singapore; [2]Department of Pathology, Tan Tock Seng Hospital, Jalan Tan Tock Seng, S308433, Singapore; [3]Department of Otolaryngology, Singapore General Hospital, Outram Road, S169608, Singapore

**Summary** The expression of metallothionein (MT), an intracellular ubiquitous low molecular weight protein thiol with antioxidant properties, was studied in nasopharyngeal cancer (NPC) and correlated with the apoptotic index. Immunohistochemical staining of randomly selected, formalin-fixed and paraffin-embedded normal and malignant nasopharyngeal tissues were analysed for the expression of MT using the commercially available E9 antibody directed against MT I and MT II isoforms. The corresponding apoptosis labelling indices were evaluated by the TUNEL method. Localization of MT at the ultrastructural level was studied by immunogold labelling. All the tumour sections (17 specimens) showed MT-immunopositivity. A direct correlation between the percentage of MT-positive cells and the staining intensity was noted ($P < 0.001$; Pearson's $r = 0.95$). There was absence of cytoplasmic staining and only nuclear staining (with localization in the nucleoplasm) was demonstrated in the tumour cells. In normal epithelium of the nasopharynx, the basal layer was stained. An inverse relationship was observed between the level of MT expression and the apoptotic index in the NPC tissues ($P = 0.0059$; Pearson's $r = –0.6380$). The results suggest that overexpression of MT in NPC may protect the tumour cells from entering into the apoptotic process and thereby contribute to tumour expansion. Preferential localization of MT in the nuclei of NPC cells may possibly enhance radioresistance since radiotherapy is known to eradicate tumour cells by free radical-induced apoptosis. © 2000 Cancer Research Campaign

**Keywords**: nasopharyngeal cancer; metallothionein immunohistochemistry; nuclear localization; apoptosis; ultrastructure

Nasopharyngeal carcinoma (NPC) is a biologically distinct form of head and neck cancer occurring more frequently in Oriental population than in whites. It is a unique head and neck cancer because of its epidemiology, association with Epstein–Barr virus and biological behaviour (Roychowdhury et al, 1996). The tumour is known to grow rapidly and is highly metastatic. Histologically, these tumours are classified by WHO into three types as squamous cell carcinoma, non-keratinizing and undifferentiated carcinoma (Shanmugaratnam et al, 1979), with the predominant histological type being poorly or undifferentiated carcinoma (Lo et al, 1992).

In an effort to understand the biology of this intriguing tumour, various biomarkers such as oncogenes, p53 tumour suppressor gene, waf1 protein, proliferating cell nuclear antigen (PCNA), telomerase, microsatellite markers and cytogenetic changes have been examined (Lo et al, 1992; Kouvidou et al, 1997; Oh and Mao, 1997). One biomarker which has recently been shown to be expressed in various human tumours but hitherto still unreported in NPC is metallothionein (MT). MTs are cysteine rich intracellular metal binding proteins that are present in a wide variety of eukaryotes (Kagi and Schaffer, 1988). Functioning as antioxidants, they also have a protective role against hydroxyl free radicals (Lazo and Pitt, 1995). This is especially relevant in NPC, a tumour known to be markedly radiosensitive and where radiotherapy (which kills cells via free-radical-induced apoptosis) is the treatment of choice (Bailet et al, 1992; Meyn et al, 1996).

*Received 17 June 1999*
*Revised 6 October 1999*
*Accepted 18 October 1999*

*Correspondence to*: BH Bay

In light of the above findings, we decided to (a) examine the level of MT expression by immunohistochemistry and its precise localization at the ultrastructural level by immunoelectron microscopy, (b) analyse the apoptotic index by terminal deoxy nucleotidyl transferase (TdT)-mediated dUTP biotin nick end labelling (TUNEL method) and (c) explore the relationship between MT expression and apoptosis in NPC tissues.

### MATERIALS AND METHODS

### Materials and patients

Twenty-one nasopharyngeal biopsies randomly selected from Tan Tock Seng Hospital and Singapore General Hospital were included in this study. Seventeen cases were confirmed to be undifferentiated carcinoma (WHO Type 3), whereas four biopsies showed normal histology of the nasopharynx. The tumour tissues were obtained from 15 men (12 Chinese, two Malay, one Thai) and two Chinese women with a mean age of 53 years (range 33–81 years). The patients presented with ear symptoms such as decreased hearing and tinnitus, nasal symptoms such as nasal discharge and epistaxis, respiratory symptoms such as blood-stained phlegm and one presented with a neck lump. Forty-one per cent of the cases (seven patients) already had cervical lymph node enlargement at the time of diagnosis.

### Methods

#### *Immunohistochemistry*

Sections were cut from paraffin-embedded tissues at a thickness of 4 μm and mounted on APES (3-aminopropyl-tri-ethoxy silane;

Sigma-3648). The immunohistochemical staining was performed using the avidin–biotin complex technique (Zelger et al, 1993). Briefly, sections were incubated overnight at 4°C using 1:600 dilution of primary antibody E9 (Dako, Copenhagen, Denmark), a conserved epitope directed against MT I and MT II isoforms, followed by incubation with the secondary antibody (biotinylated rabbit anti-mouse immunoglobulin; Dako) at 1:200 dilution for 1 h. After a 1-h incubation with biotin and avidin–peroxidase complexes, immunostaining was demonstrated using 3,3´ diaminobenzidine tetrachloride (DAB). After immunostaining, sections were counterstained with Harris haematoxylin for 15 s, dehydrated, cleared in xylene and mounted with permount. For negative controls, tissue sections were incubated with mouse immunoglobulin of the same sub-class. For positive controls, sections from an MT positive breast cancer was used (Jin et al, 1999).

### *Analysis of immunohistochemical reactions*

For assessing the percentage of positively stained cells, sections were analysed under a light microscope (Zeiss, Axioplan) using a 40× objective. The number of positive cells per high power field was determined in relation to the total number of cells in that field. A total of ten high power fields were randomly chosen. The relative intensity of staining was assessed subjectively by two members of the group. The immunohistochemical staining was graded as '+' for weak staining, '++' for moderate staining and '+++' for strong staining. To study the correlation between the percentage of MT-positive cells and staining intensity, a numerical scoring was also instituted for staining intensity: 1 for '+', 2 for '++' and 3 for '+++'.

### *Transmission electron microscopy and immunoelectron microscopy*

Tissue from a confirmed undifferentiated NPC (by light microscopy) was fixed in 3% glutaraldehyde and 2% paraformaldehyde before osmication in 2% osmium tetroxide as previously described (Bay et al, 1998). Post-osmicated samples were dehydrated in an ascending series of ethanol and embedded in epoxy resin. Ultrathin sections for immunolabelling and conventional electron microscopy were cut and mounted on formvar-coated nickel grids. For unmasking of antigens, the procedure of Stirling and Graff (1995) was followed. Briefly, grids for immunolabelling were incubated on large drops of saturated aqueous solution of sodium metaperiodate for 1 h at room temperature (RT) in a humidified chamber followed by washing in distilled water for 45–60 s. The grids were then heated by floating them sections down in preheated 0.01 M sodium citrate buffer (pH 6) maintained at 95–100°C for 10 min. The sections were allowed to cool in the retrieval medium for 15 min followed by washing in distilled water for 60 s. After antigen unmasking treatment, grids were first incubated on 0.01 M phosphate-buffered saline (PBS) pH 7.4 containing 1% immunoglobulin-free bovine serum albumin (BSA) for 10 min. Sections were then incubated overnight (18 h) at 4°C with the E9 primary antibody at a dilution of 1:100 in PBS containing 1% BSA and 1% Tween-20. The grids were washed by floating them on drops of PBS (3 changes, 5 min each), followed by 5-min incubation on PBS with 1% BSA. Bound antibodies were visualized by incubating the sections for 1 h on EM goat anti-mouse immunoglobulin gold conjugate (20 nm) diluted 1:100 in PBS with 1% BSA and 1% Tween-20 at RT before washing with water (3 changes, 5 min each). The grids were then stained with 1% aqueous uranyl acetate and lead citrate. The sections were examined in Philips 120 EM transmission electron microscope. Immunogold specificity was tested by omitting the primary antibody and incubating the sections with antibody diluent.

### *In situ detection of apoptotic cells in paraffin sections (TUNEL method)*

Apoptotic cells in formalin-fixed and paraffin-embedded tissue sections were detected by the commercially available Tdt Frag EL[tm] DNA Fragmentation Detection kit (Oncogene Research Products, Cambridge, MA, USA). Briefly paraffin sections were dewaxed in xylene and rehydrated in alchohol, followed by incubation with proteinase k (20 µg ml$^{-1}$) at RT for 20 min. Endogenous peroxidase activity was blocked by incubating the slides in 3% hydrogen peroxide. Following the application of the equilibration buffer, the sections were incubated in working strength TdT enzymes (containing dUTP–digoxigenin) at 37°C for 90 min. The reaction was stopped by a prewarmed working strength stop buffer. After washing in distilled water an anti-digoxigenin antibody fragment carrying enzyme peroxidase was applied in a humidified chamber for 30 min at RT. The localized peroxidase enzyme catalytically generated an intense signal from the chromogenic DAB substrate. The sections were counterstained with methyl green. Quantitative evaluation of positive apoptotic cells was done by examining the sections in ten random regions and counting the number of TUNEL-positive cancer cells among 1000 carcinoma cells using the Zeiss Axioplan microscope. The apoptotic index was expressed as positive cells per 100 cancer cells.

### *Statistical analyses*

The Graphpad Prism software package was used for statistical analysis. The relationship between the percentage of MT-immunostained cells and cervical lymph node status was analysed by $\chi^2$ test. Pearson's correlation was used to analyse the relationship between the percentage of MT-positive cells with age, staining intensity and apoptosis.

### RESULTS

### MT immunohistochemistry

MT-immunopositivity was detected in all the 17 NPC tissue sections examined. An example of an undifferentiated NPC tissue section (Figure 1A) exhibiting high levels of MT expression is shown in Figure 1B. MT staining was localized to the nucleus and absent in the cytoplasm. Generally, in sections with high MT-immunopositivity, the immunopositive cells formed contiguous foci or sheets. In sections with low MT immunoreactivity, scattered positive cells predominate and on some occasions isolated clusters of positive cells were seen. The four tissues with normal histology of the nasopharynx showed MT staining in the inner layer of the epithelium, the germinal centers and the lining vascular endothelium.

The percentage of MT-immunopositive tumour cells determined under light microscopy ranged from 3% to 57.9% (Table 1). In nine of the 17 cases, the tumour sections exhibited more than 10% MT-immunopositivity. With regard to staining intensity, tumour sections with weakly stained cells formed the largest group (47%).

© 2000 Cancer Research Campaign

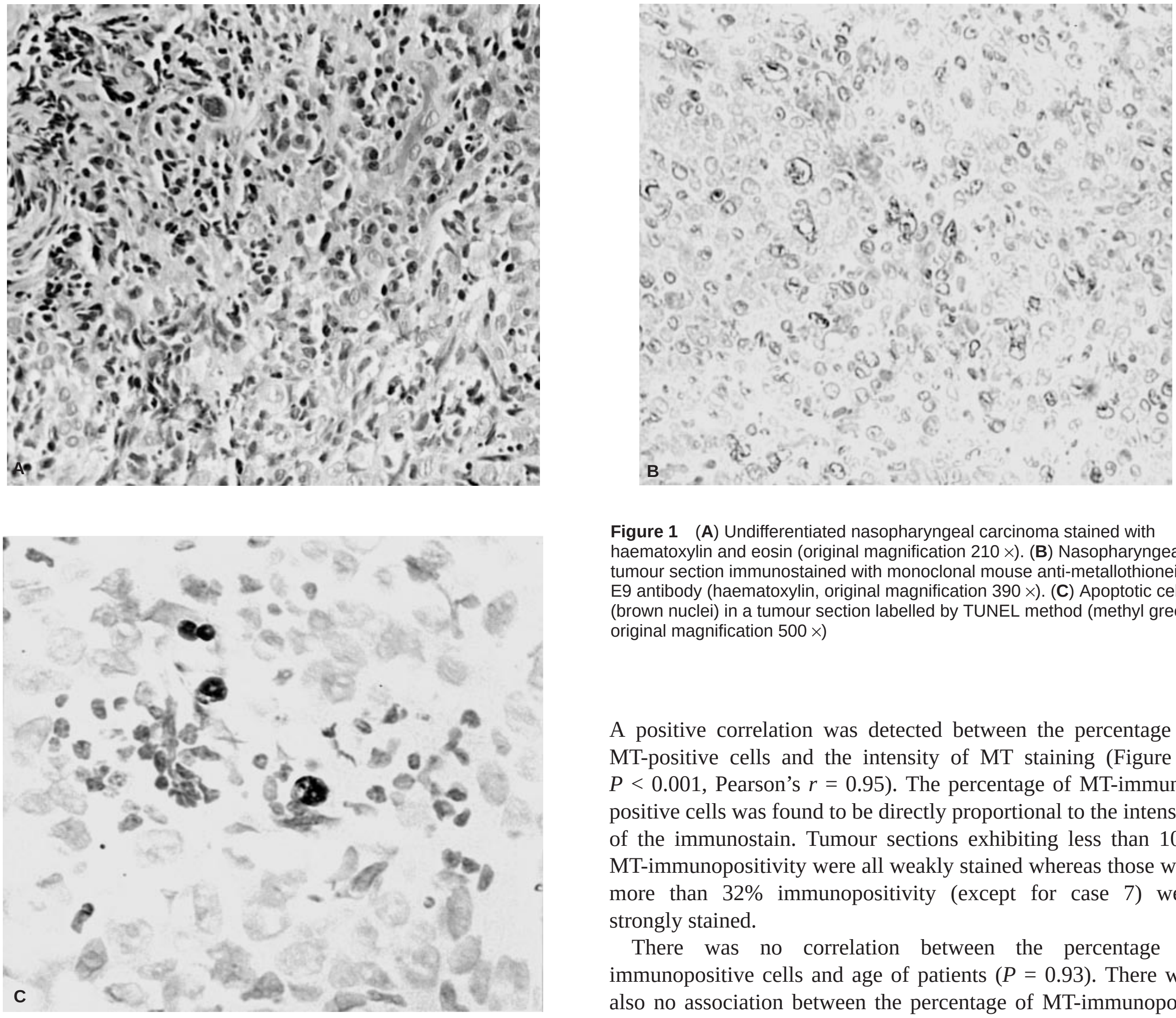

**Figure 1** (**A**) Undifferentiated nasopharyngeal carcinoma stained with haematoxylin and eosin (original magnification 210 ×). (**B**) Nasopharyngeal tumour section immunostained with monoclonal mouse anti-metallothionein E9 antibody (haematoxylin, original magnification 390 ×). (**C**) Apoptotic cells (brown nuclei) in a tumour section labelled by TUNEL method (methyl green, original magnification 500 ×)

**Table 1** Metallothoinein immunoreactivity and corresponding apoptotic rates in nasopharyngeal cancers

| Patient | MT immunoreactivity (% stained cells) | MT staining intensity | Apoptotic index (positive cells/100 cancer cells) |
|---|---|---|---|
| 1 | 57.2 | +++ | 0.3 |
| 2 | 43.5 | +++ | 0.2 |
| 3 | 57.9 | +++ | 0.1 |
| 4 | 23.8 | ++ | 0.4 |
| 5 | 18.3 | ++ | 0.3 |
| 6 | 32.4 | +++ | 0.5 |
| 7 | 33.4 | ++ | 0.6 |
| 8 | 5.5 | + | 0.8 |
| 9 | 5.0 | + | 1.0 |
| 10 | 5.3 | + | 0.7 |
| 11 | 4.6 | + | 1.0 |
| 12 | 3.8 | + | 1.8 |
| 13 | 4.1 | + | 0.6 |
| 14 | 3.0 | + | 0.5 |
| 15 | 3.4 | + | 0.7 |
| 16 | 8.6 | + | 0.6 |
| 17 | 4.9 | + | 0.7 |

A positive correlation was detected between the percentage of MT-positive cells and the intensity of MT staining (Figure 2; $P < 0.001$, Pearson's $r = 0.95$). The percentage of MT-immunopositive cells was found to be directly proportional to the intensity of the immunostain. Tumour sections exhibiting less than 10% MT-immunopositivity were all weakly stained whereas those with more than 32% immunopositivity (except for case 7) were strongly stained.

There was no correlation between the percentage of immunopositive cells and age of patients ($P = 0.93$). There was also no association between the percentage of MT-immunopositive cells (subdivided into two groups: > 10% and < 10% MT-immunopositive cells) and the cervical lymph node status ($P = 0.20$).

### Ultrastructural localization of MT

Under transmission electron microscopy, the cancer cells are characterized by the presence of (a) desmosomes (macula adherens), which are specialized junctional membranes at the contiguous border of adjacent tumour cells, (b) abundant tonofilaments (keratin fibrils) in the cytoplasm and (c) large nuclei (with high nucleocytoplasmic ratio) bounded by a nuclear membrane with undulating margins (Figure 3A). The MT protein was localized essentially in the nucleoplasm of the tumour cell nuclei rather than in the nuclear membrane or cytoplasm (Figure 3B).

### Apoptosis

The apoptotic index ranged from 0.1 to 1.8 cells per 100 cancer cells (Table 1). An example of apoptotic cells in a tumour section is shown in Figure 1C. The percentage of MT-immunopositive cells was found to be inversely proportional to the apoptotic rate (Figure 4; $P < 0.001$, Pearsons $r = –0.6380$).

 *© 2000 Cancer Research Campaign*

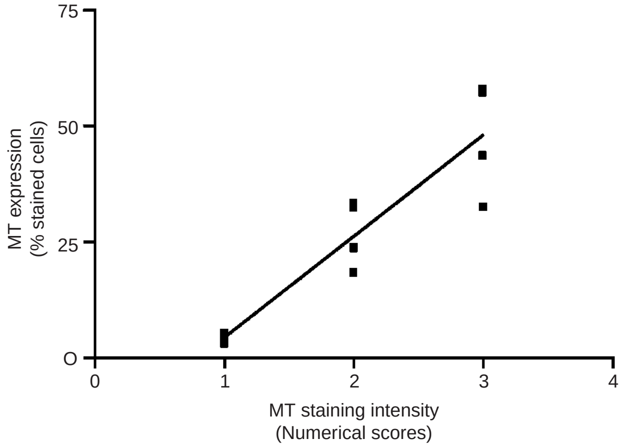

**Figure 2** Positive correlation between MT-immunopositive cells (expressed as percentage of stained cells) with the staining intensity ($r$ = 0.95, $r^2$ = 0.9025, $P$ < 0.001)

### DISCUSSION

It is well established that MT, a superfamily of metal-binding macromolecules, participates in sulphur-based metal clusters (Kagi and Schaffer, 1988). In fact, a conspicuous feature of all the different subclasses of mammalian MTs is the presence of cysteine residues which have the capability to bind metals. Consequently, MTs have metalloregulatory functions in cell growth and differentiation (Cherian et al, 1994). The ubiquitous occurrence of MT and the conservation of its structure further suggests that besides having a specific role in normal cellular metabolism, MTs may be involved pleiotropically in a plethora of different biological functions (Kagi and Schaffer, 1988).

Since MT is known to possess antioxidant properties which protect cells from free radical-induced apoptosis (Lazo and Pitt, 1995), its possible role in carcinogenesis has attracted attention in recent years. Amongst others, overexpression of MT has been reported in testicular tumours (Kontozoglou et al, 1989), bladder carcinomas (Bahnson et al, 1991), ovarian tumours (Germain et al, 1996; Tan et al, 1999), malignant melanoma (Zelger et al, 1993) and invasive breast cancers (Schmid et al, 1993; Jin et al, 1999). In tumours such as ductal breast cancer and malignant melanoma, MT expression is associated with more malignant and higher grade tumours. In head and neck cancers, MT expression in squamous cell carcinoma of the tongue has been suggested to delay cells entering apoptosis (Sundelin et al, 1997). The present study is the first to document the overexpression of MT in NPC. All the tumour specimens exhibited MT-immunopositivity and tumour sections with higher percentages of MT-immunopositive cells showed more intense staining. The methodology for detecting MT protein includes Western blotting, electrophoresis followed by silver staining and immunohistochemistry (Aoki and Suzuki, 1991; McCormick and Lin, 1991; Zelger et al, 1993). We chose to investigate MT expression in NPC by immunohistochemical staining as immunohistochemical detection has the advantage of correlating MT protein expression with the pathology of the tumour.

The MT immunoreactivity was found to be mainly localized to the nucleus, in particular the nucleoplasm. It is possible for the MT protein to diffuse through the nuclear pore complex either by passive diffusion or via an active, signal-mediated process (Woo et al, 1996). Another possible reason for the preferential localization

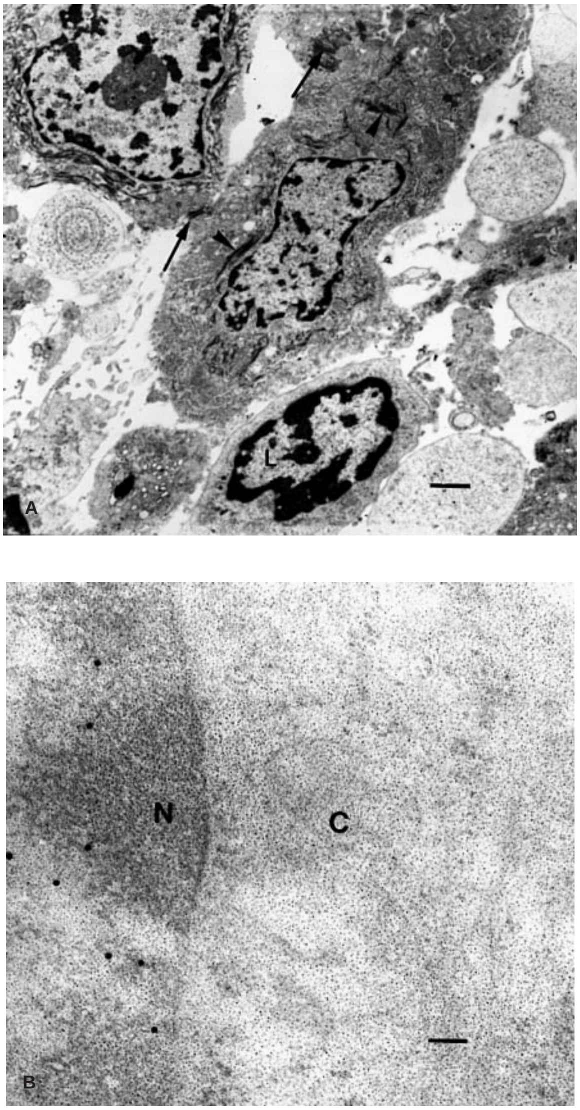

**Figure 3** (**A**) Transmission electron micrograph of NPC cells typified by desmosomes (arrows) and tonofilaments (arrowheads). A lymphocyte (L) is seen in the vicinity (original magnification, 7260 ×). Scale bar = 1 µm. (**B**) Immunogold particles localized mainly in the nucleoplasm of tumour cell nucleus (N) and not in the cytoplasm (C) (original magnification, 40 700 ×). Scale bar = 100 nm

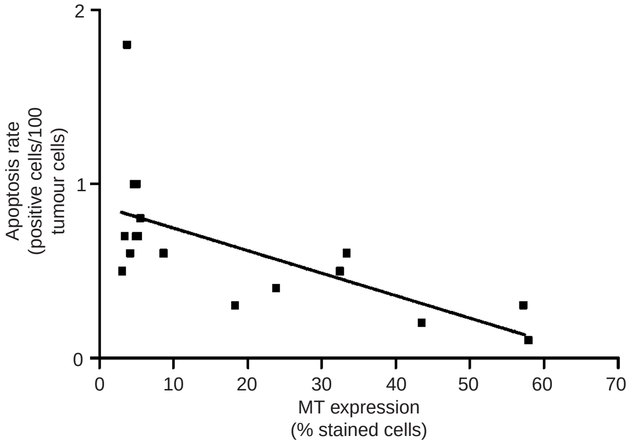

**Figure 4** Inverse relationship between MT immunopositive cells (expressed as percentage of stained cells) with the apoptotic index ($r$ = –0.6380; $^2$ = 0.4070; $P$ = 0.0059)

of MT in the nucleus could be the interaction of MT with zinc ions (Jin et al, 1999), which are known to be raised in NPC (Bay et al, 1997). Since the nuclear phenotype has been reported in human malignancies typified by rapid growth (Woo et al, 1996), such as bladder tumour cells (Kuo et al 1994), it is also possible that over-expression of MT in the nuclei of NPC cells is related to high proliferative activity.

Antioxidants are known to restrict the apoptotic process in many systems (Briehl and Baker, 1996). In this regard, modulation of the cellular antioxidant defence would have far reaching effects on tumour response to apoptotic stimuli although reports have been varied. Down-regulation of MT with increased apoptosis has been observed in hepatocellular carcinoma (Cai et al, 1998), whereas increased incidence of apoptosis with increasing immunoreactivity of MT has been reported in renal cell carcinoma (Zhang and Takenaka, 1998). In the present study, we found an inverse correlation in tumour cells exhibiting high MT expression and the rate of apoptosis. Our results appear to indicate that MT overexpression may contribute to the rapid growth of these tumours by retarding apoptosis.

In addition, support for the hypothesis that MTs participate in intracellular defence against reactive oxygen species is derived from the observation that MT genes are transcriptionally activated in cells and tissues during oxidative stress (Lazo and Pitt, 1995). Endogenous antioxidants are known to protect cells from reactive oxygen species mediated DNA damage (occurring in the nucleus) as a result of radiation (Buttke and Sandstorm, 1994). Hence, the radioprotective effect of MT against DNA damage by free radicals is likely to be enhanced by nuclear sequestration. The demonstration of nuclear localization of MT would certainly not augur well for NPC patients who have a high percentage of MT-positive tumour cells. Besides protecting the cell against apoptosis, the abundant nucleophilic sulphydryl groups in MT could interact with many electrophilic toxins, thereby making them attractive candidates as modulators of cellular senstivity to electrophilic anticancer agents. In fact, induction of MT has also been suggested to be an important mechanism in potentiating the resistance of tumour cells to chemotherapeutic agents (Kelley et al, 1988).

In conclusion, we have demonstrated that MT is overexpressed in NPC by immunohistochemical staining. The unique features of the immunostaining are: (a) preferential localization of MT in the nucleus, (b) a positive correlation between the percentage of MT immunopositive cells and the intensity of staining, (c) an inverse correlation between MT expression and apoptosis favouring the hypothesis that MT protects the tumour cells from undergoing apoptosis and contributes to tumour expansion. Furthermore, up-regulation of MT in NPC tissues may indeed be important in the development of radioresistance in NPC. To elucidate whether the immunohistochemical observation of MT overexpression can be used as a marker of radioresistance in nasopharyngeal cancers, a prospective investigation entailing the retrospective analysis of archival material from NPC patients, will be carried out.

### ACKNOWLEDGEMENTS

The authors thank M Sim and TY Yick for technical assistance. This work was supported by a grant from the National Medical Research Council (Singapore) (NMRC 0244/97).

### REFERENCES

Aoki Y and Suzuki KT (1991) Detection of metallothionein by Western blotting. *Methods Enzymol* **205**: 108–114

Bahnson RR, Banner BF, Ernstoff MS, Lazo JS and Cherian MG (1991) Immunohistochemical localisation of metallothionien in transitional cell carcinoma of the bladder. *J Urol* **146**: 1518–1520

Bailet JW, Mark RF, Abemayor E, Lee SP, Tran LM, Julliard G and Ward PH (1992) Nasopharyngeal carcinoma: treatment results with primary radiation therapy. *Laryngoscope* **102**: 965–972

Bay BH, Chan YG, Fong CM and Leong HK (1997) Differential cellular zinc levels in metastatic and primary nasopharyngeal carcinoma. *Int J Oncol* **11**: 745–748

Bay BH, Chan YG, Tuck YK and Leong HK (1998) Electron microscopic observations and X-ray microanalysis of a multinucleated giant cell. *J Electron Microsc* **47**: 359–361

Briehl MM and Baker AF (1996) Modulation of the antioxidant defence as a factor in apoptosis. *Cell Death Differ* **3**: 63–70

Buttke TM and Sandstrom PA (1994) Oxidative stress as a mediator of apoptosis. *Immunol Today* **15**: 7–10

Cai L, Wang GJ, Xu ZL, Deng DX, Chakrabarti S and Cherian MG (1998) Metallothionein and apoptosis in primary human hepatocellular carcinoma from Northern China. *Anticancer Res* **18**: 4667–4672

Cherian MG, Howell SB, Imura N, Klaassen CD, Koropatnick J, Lazo JS and Waalkes MP (1994) Role of metallothionein in carcinogenesis. *Toxicol Applied Pharmacol* **126**: 1–5

Germain I, Tetu B, Brisson J, Mondor M and Cherian MG (1996) Markers of chemoresistance in ovarian carcinomas: an immunohistochemical study of 86 cases. *Int J Gynecol Pathol* **15**: 54–62

Jin R, Bay BH, Tan PH and Tan BKH (1999) Metallothionein expression and zinc levels in invasive ductal carcinoma of the breast. *Oncol Rep* **6**: 871–875

Kagi JHR and Schaffer A (1988) Biochemistry of metallothionein. *Biochemistry* **27**: 8509–8514

Kelley SL, Basu A, Teicher BA, Hacker P, Hamer DH and Lazo JS (1988) Overexpression of metallothionein confers resistance to anticancer drugs. *Science* **241**: 1813–1815

Kontozoglou TE, Banerjee D and Cherian MG (1989) Immunohistochemical localisation of metallothionein in human testicular embryonal adenocarcinoma. *Virchows Arch* **45**: 545–549

Kouvidou CH, Stefanaki K, Dai Y, Tzardi M, Koutsoubi K, Darivianaki K, Karidi E, Rontogianni D, Zois E, Kakolyris S, Georgoulias V, Delides G and Kanavaros P (1997) P21/waf1 protein expression in nasopharyngeal carcinoma. Comparitive study with PCNA, P53 and MDM-2 protein expression. *Anticancer Res* **17**: 2615–2620

Kuo SM, Kondo Y, DeFilippo JM, Ernstoff MS, Bahnson RR and Lazo JS (1994) Subcellular localization of metallothionein IIA in human bladder tumor cells using a novel epitope-specific antiserum. *Toxicol Applied Pharmacol* **125**: 104–110

Lazo JS and Pitt BR (1995) Metallothionein and cell death by anticancer drugs. *Annu Rev Pharmacol Toxicol* **35**: 635–653

Lo KW, Mok CH, Huang DP, Liu YX, Choi PH-K, Lee JCK and Tsao SW (1992) P53 mutation in human nasopharyngeal carcinomas. *Anticancer Res* **12**: 1957–1964

McCormick CC and Lin LY (1991) Quantification and identification of metallothioneins by gel electrophoresis and silver staining. *Meth Enzymol* **205**: 71–78

Meyn RE, Stephens LC and Milas L (1996) Programmed cell death and radioresistance. *Cancer Metastasis Rev* **15**: 119–131

Oh Y and Mao L (1997) Biomarkers in head and neck carcinomas. *Curr Opin Oncol* **9**: 247–256

Roychowdhury DF, Tseng A Jr, Fu KK, Weinberg V and Weidner N (1996) New prognostic factors in nasopharyngeal carcinoma. *Cancer* **77**: 1419–1426

Schmid KW, Ellis IO, Gee JM, Darke BM, Lees WE, Kay J, Cryer A, Stark JM, Ofner D, Dunser M, Margreiter R, Daxenbichter G, Nicholson RI, Bier B, Bocker W and Jasani B (1993) Presence and possible significance of immunocytochemically demonstrable metallothionein overexpression in primary invasive ductal carcinoma of the breast. *Virchows Arch* **422**: 153–159

Shanmugaratnam K, Chan SH, de-The G, Goh EH, Khor TH, Simons MJ and Tye CY (1979) Histopathology of nasopharyngeal carcinoma. *Cancer* **44**: 1029–1044

Stirling JW and Graff PS (1995) Antigen unmasking for immunoelectron microscopy: labelling is improved by treating with sodium ethoxide or sodium

 © 2000 Cancer Research Campaign metaperiodate, then heating on retrieval medium. *J Histochem Cytochem* **43**: 115–123

Sundelin K, Jadner M, Norberg-Spaak L, Davidsson A and Hellquist HB (1997) Metallothionein and Fas (CD95) are expressed in squamous carcinoma of the tongue. *Eur J Cancer* **33**: 1861–1864

Tan Y, Sinniah R, Bay B-H and Singh G (1999) Metallothionein expression and nuclear size in benign, borderline and malignant serous ovarian tumors. *J Pathol* **189**: 60–65

Woo ES, Kondo Y, Watkins SC, Hoyt DG and Lazo JS (1996) Nucleophilic distribution of metallothionein in human tumor cells. *Exp Cell Res* **224**: 365–371

Zhang X-H and Takenaka I (1998) Incidence of apoptosis and metallothionein expression in renal cell carcinoma. *Br J Urol* **81**: 9–13

Zelger B, Hittmair A, Schir M, Ofner C, Ofner D, Fritsch PO, Bocker W, Jasani B and Schmid KW (1993) Immunohistochemically demonstrated metallothionein expression in malignant melanoma. *Histopathology* **23**: 257–264

© 2000 Cancer Research Campaign
